# Plasmid carriage can limit bacteria–phage coevolution

**DOI:** 10.1098/rsbl.2015.0361

**Published:** 2015-08

**Authors:** Ellie Harrison, Julie Truman, Rosanna Wright, Andrew J. Spiers, Steve Paterson, Michael A. Brockhurst

**Affiliations:** 1 Department of Biology, University of York, York YO10 5DD, UK; 2 Institute of Integrative Biology, University of Liverpool, Liverpool L69 7ZB, UK; 3 SIMBIOSIS Centre, University of Abertay, Dundee DD1 1HG, UK

**Keywords:** bacteria–phage coevolution, conjugative plasmid, mucoid conversion

## Abstract

Coevolution with bacteriophages is a major selective force shaping bacterial populations and communities. A variety of both environmental and genetic factors has been shown to influence the mode and tempo of bacteria–phage coevolution. Here, we test the effects that carriage of a large conjugative plasmid, pQBR103, had on antagonistic coevolution between the bacterium *Pseudomonas fluorescens* and its phage, SBW25*ϕ*2. Plasmid carriage limited bacteria–phage coevolution; bacteria evolved lower phage-resistance and phages evolved lower infectivity in plasmid-carrying compared with plasmid-free populations. These differences were not explained by effects of plasmid carriage on the costs of phage resistance mutations. Surprisingly, in the presence of phages, plasmid carriage resulted in the evolution of high frequencies of mucoid bacterial colonies. Mucoidy can provide weak partial resistance against SBW25*ϕ*2, which may have limited selection for qualitative resistance mutations in our experiments. Taken together, our results suggest that plasmids can have evolutionary consequences for bacteria that go beyond the direct phenotypic effects of their accessory gene cargo.

## 1. Introduction

Lytic phages are abundant in natural environments and a major cause of bacterial mortality [1]. It is increasingly recognized that bacteria–phage coevolution, the reciprocal evolution of bacterial resistance and phage infectivity, is an important evolutionary process shaping microbial communities [2,3]. Many factors, both environmental and genetic, have been shown to affect this process [4]. For example, both the mode and tempo of coevolution is strongly dependent on factors affecting genetic variation or the strength of reciprocal selection, such as mutation rate [5], population mixing [6–8], or resource availability [9,10]. Environmental variables can also lead to qualitative differences in the evolutionary response to phage infection, for instance favouring different forms of resistance in different environments [6,11]. In addition, bacterial genetic background can affect the outcome of bacteria–phage coevolution: for example, epistatic interactions between the costs of deleterious mutations and phage resistance mutations can constrain the rate of bacterial resistance evolution and thereby limit the rate of coevolution [12].

Conjugative plasmids, like phages, are ubiquitous in bacterial populations and drive bacterial genomic diversity through horizontal gene transfer [13]. While the accessory genes carried on plasmids can be highly beneficial to bacteria in some environments, plasmid acquisition represents a major change to bacterial genomic content, leading to biosynthetic costs and cellular disruption [14,15]. Furthermore, plasmid acquisition can increase the vulnerability of bacterial cells to environmental stressors [16]. Plasmid carriage is therefore likely to impact upon bacteria–phage coevolution, but this possibility, to our knowledge remains untested. We explored this using experimental coevolution of laboratory communities of the lytic phage SBW25*ϕ*2 and its host bacterium *Pseudomonas fluorescens* SBW25 either with or without the plasmid pQBR103; a conjugative 425Kb mercury resistance plasmid [17] isolated from the same soil community as SBW25 [18]. Following c.130 bacterial generations of coevolution, we assessed the relative bacterial resistance and phage infectivity phenotypes that evolved in each treatment using a cross-infection assay.

## 2. Methods

### (a) Strains and culture conditions

Populations of *P. fluorescens* SBW25-Gm with or without plasmid pQBR103 [19] were initiated from single clones. Six replicate populations were established for each of four factorial combinations of plasmid (with or without) and phage (with or without) treatments. All populations were founded with approximately 10^8^ bacterial cells plus approximately 10^6^ SBW25*ϕ*2 particles in phage-containing treatments, and cultured in 30 ml microcosms containing 6 ml of King's broth (KB) supplemented with 8 µM HgCl_2_ to ensure retention of the plasmid [19]. Preliminary experiments showed that at 8 µM HgCl_2_ there was no significant difference in growth between plasmid-containing and plasmid-free cultures (electronic supplementary material, figure S1). Populations were incubated at 28°C, shaken at 180 rpm and propagated by transferring 1% to fresh media every 48 h for 20 transfers. Bacteria and phages were plated at every fourth transfer to measure phage and bacterial density, plasmid prevalence [19] and colony morphology [20] (electronic supplementary material, figure S2).

### (b) Measuring bacterial resistance/phage infectivity

Resistance/infectivity was measured as the Reduction in Bacterial Growth (RBG) associated with phage co-culture (adapted from Poullain *et al.* [21]). Phage populations and 20 randomly picked bacterial clones were isolated from each phage containing population at transfer 20. Bacterial clones were then grown up in 150 µl KB in 96-well plates either alone or in the presence of each of the 12 phages populations. Cultures were incubated at 28°C and density measured at 0 and 20 h growth. RBG values were estimated for each interaction as3.1
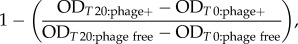


where OD stands for optical density at 600 nm. RBG estimates for the ancestral strains show that both ancestral plasmid-free and plasmid-containing strains were highly susceptible to ancestral phage infection (electronic supplementary material, figure S3).

### (c) Estimating epistasis between costs of plasmid carriage and phage resistance

To test for an effect of plasmid carriage on the costs of phage resistance mutations, competitive fitness assays of eight spontaneous phage resistance mutants [19] and the phage-sensitive ancestor with and without the plasmid were performed in triplicate. Overnight cultures of each strain were mixed 1 : 1 with an isogenic lacZ-marked *P. fluorescens* SBW25 and inoculated into 6 ml of KB supplemented with 8 µM HgCl_2_ and grown for 48 h. Samples were plated at 0 and 48 h onto KB agar supplemented with X-gal, and relative fitness was calculated as the ratio of Malthusian parameters of competing strains [22].

## 3. Results and discussion

**Figure 1.**
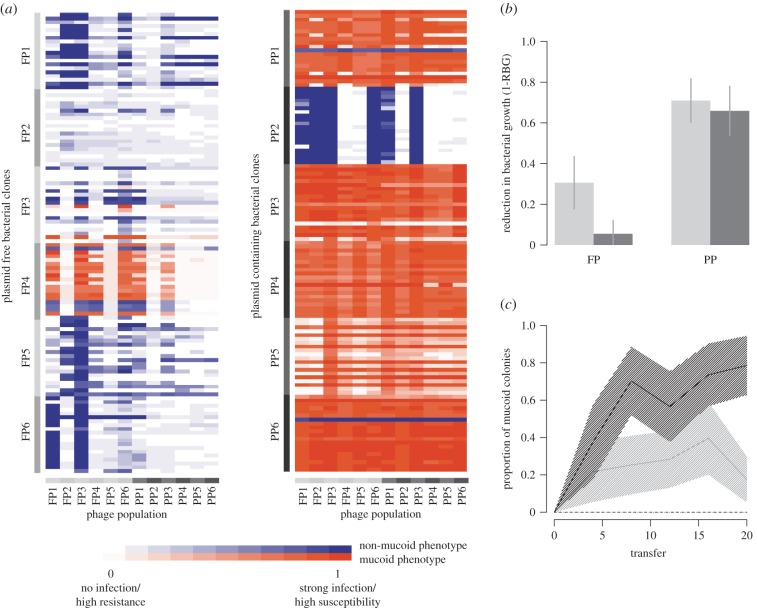
Bacterial responses to phage infection. (*a*) Infection heat maps of pairwise interactions between the 120 bacterial clones (20 per population, six populations per treatment) from the plasmid-free (FP1-FP6; left) and plasmid-containing (PP1-PP6; right) populations with phage populations from both treatments (*n* = 12). Bacterial clones are grouped by population along the *y*-axis and phage populations grouped by treatment along the *x*-axis, indicated by grey tabs. Colours denote the mucoid status of each clone with intensity scaled by 1-RBG value (where darker indicates high 1-RBG and therefore phage infection). (*b*) Mean reduction in bacterial growth owing to phage predation (1-RBG) for bacterial clones from the plasmid-free (FP) and plasmid-containing (PP) treatments challenged against phages isolated from the plasmid-free (light) and plasmid-containing (dark) treatments. Lines show standard error of population means (*n* = 6). (*c*) Mean frequency of mucoidy over time. Lines show means (*n* = 6) for the four treatments; plasmid-containing treatments are shown in black and plasmid-free in grey. Phage-containing treatments are shown as fixed lines and phage-free control lines shown as dashed. Shading indicates standard error.

Plasmid carriage constrained both the evolution of bacterial resistance and phage infectivity. Plasmid-free bacteria evolved significantly higher rates of phage resistance compared with plasmid-carrying bacteria (figure 1*a,b*; PLASMID TREATMENT_BACTERIA_ *χ*^2^ = 166.5, *p* < 0.0001). Phage infectivity, however, was dependent on which treatment bacteria were isolated from (PLASMID TREATMENT_BACTERIA_ × PLASMID TREATMENT_PHAGE_ *χ*^2^ = 85.5, *p* < 0.0001). When challenged against plasmid-carrying bacteria, phages from both treatments were equally infectious (PLASMID TREATMENT_PHAGE_ *χ*^2^ = 0.546, *p* = 0.46). However, against plasmid-free bacteria, phages from the plasmid-containing treatment were significantly less infectious than phages from the plasmid-free treatment (PLASMID TREATMENT_PHAGE_ *χ*^2^ = 6.62, *p* = 0.0101).

Our results therefore suggest that the carrying pQBR103 constrained bacterial resistance evolution, which in turn weakened selection for phage infectivity. One possible explanation for this is that plasmid carriage exacerbated the cost of phage resistance mutations, making resistance disproportionately costly and thereby limiting bacterial evolution [12]. To test this hypothesis, we measured the fitness of eight spontaneous phage-resistant mutants with and without the plasmid. In the ancestral SBW25 background, the plasmid did not significantly alter bacterial fitness (*t*_2.12_ = −0.026, *p* = 0.982). This was expected as experiments were conducted in mercury-supplemented media to ensure plasmid retention. In the phage-resistant backgrounds, we observed no evidence that plasmid-carriage affected the cost of phage resistance (electronic supplementary material, figure S4; for each clone *p* > 0.1). Indeed, in all but one case, plasmid-carriage appeared to alleviate the cost of phage resistance although this was significant in only two clones (A2 *t*_3.98_ = −3.201, *p* = 0.033 and E5 *t*_3.226_ = −5.649, *p* = 0.009). It is unlikely therefore that negative epistatic interactions constrained bacteria–phage coevolution in our experiment.

Surprisingly, we observed significant effects of the plasmid on bacterial colony morphology. In the presence of phages, bacteria evolved a mucoid colony morphology [11,20] (*z* = 30.83, *p* < 0.0001), with far higher mucoidy frequencies among plasmid-carrying compared to plasmid-free populations exposed to phage (figure 1*c*; *z* = 4.473, *p* < 0.0001). Whereas mucoidy transiently appeared in five out of six plasmid-free populations, [20,23], mucoidy approached fixation in five out of six replicates (electronic supplementary material, figure S2). Transient mucoidy in the plasmid-free populations is consistent with previous studies of this bacteria–phage system [24]: without plasmids, mucoidy occurs in response to phages but rarely reaches high frequencies except under restricted culturing conditions [20,23]. Together this suggests that the emergence of the mucoid phenotype was not directly linked to plasmid carriage, for instance, owing to specific plasmid-encoded genes, but that plasmid carriage and phage attack interacted to select for the evolution of mucoidy in *P. fluorescens*.

Mucoidy is caused by over-production of alginate and provides partial resistance to phages in this and other bacteria–phage interactions [20,23,25,26]. Thus, it appears likely that mucoidy may have evolved in place of qualitative (all-or-nothing) resistance in plasmid-carrying bacteria, and this in turn weakened selection for the evolution of qualitative resistance. Mucoidy is thought to act as a global stress response to varied environmental pressures in *Pseudomonads* [27–29] and is an important virulence factor in human chronic lung infections [30,31]. Our findings suggest that combined exposure to both phages [32] and plasmids [33] in *Pseudomonas* chronic infections could exacerbate selection for mucoidy, hastening the onset of mucoid conversion and potentially worsening patient health, raising concerns about use of phage therapy in such infections.

These data add to a growing appreciation that plasmid carriage can have complex effects on the bacterial phenotype: plasmids have been shown to alter biofilm formation [16,34], cell hydrophicity [35], tolerance to stress and motility [16]. We show that plasmid carriage can also alter biotic interactions with phages, limiting bacteria–phage coevolution and altering the longer-term evolutionary trajectory of bacterial populations.

## Supplementary Material

Fig. S1

## Supplementary Material

Fig. S2

## Supplementary Material

Fig. S3

## Supplementary Material

Fig. S4

## Supplementary Material

BL_supplimentary material.docx

## Data accessibility

Data are available from Dryad: doi:10.5061/dryad.4622g.
