# Supplementary figures and images for: Plasmid carriage can limit bacteria–phage coevolution
Source: Biol Lett. 2015 Aug;11(8):20150361. doi: 10.1098/rsbl.2015.0361 (PMC4571675; doi:10.1098/rsbl.2015.0361)

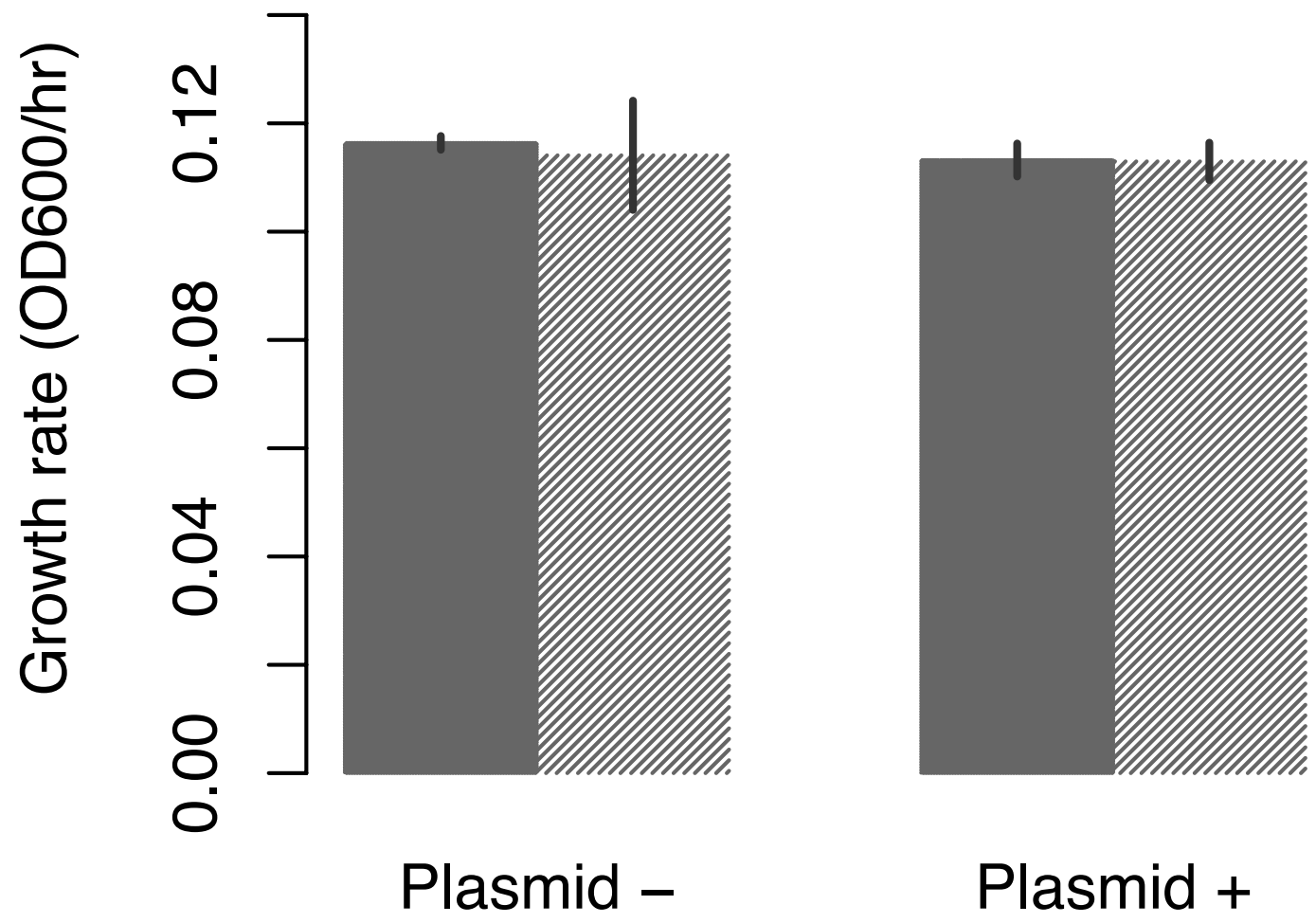

Supplement: Fig. S1 [file rsbl20150361supp1.pdf]

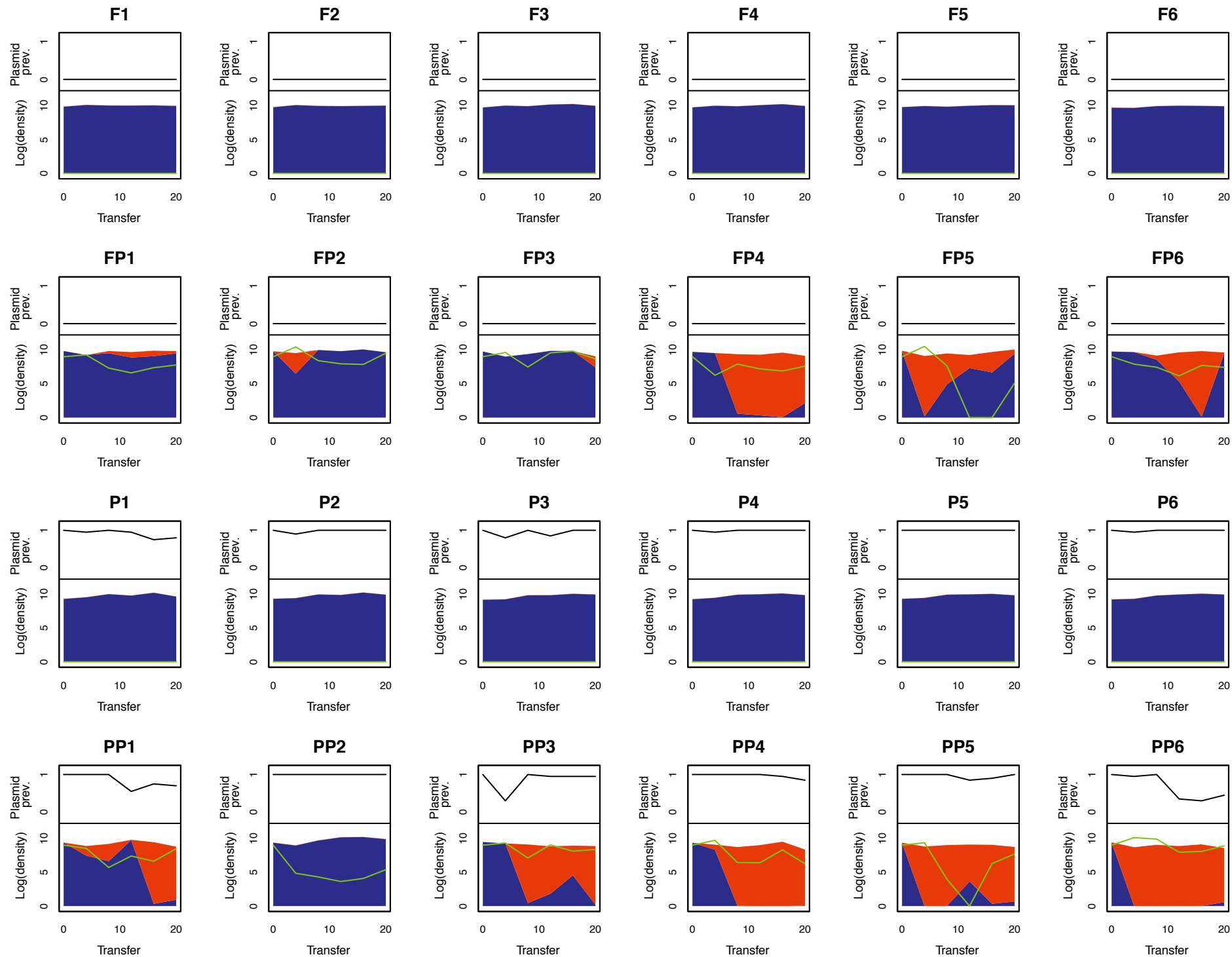

Supplement: Fig. S2 [file rsbl20150361supp2.pdf]

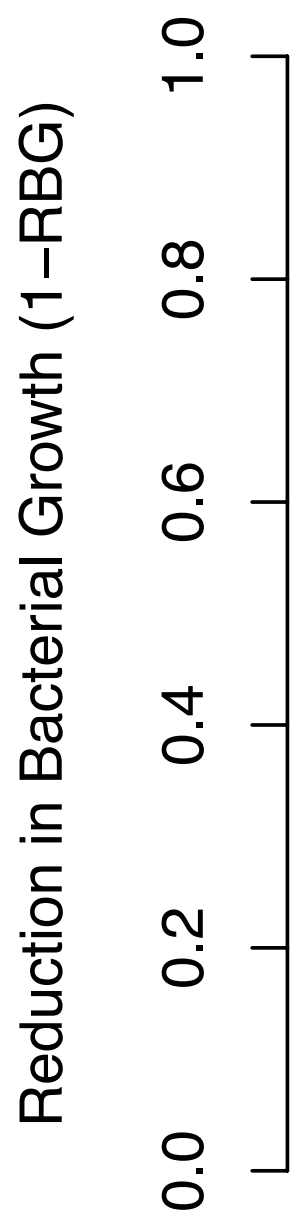

Plasmid -

Plasmid +

Supplement: Fig. S3 [file rsbl20150361supp3.pdf]

Fitness relative to plasmid free

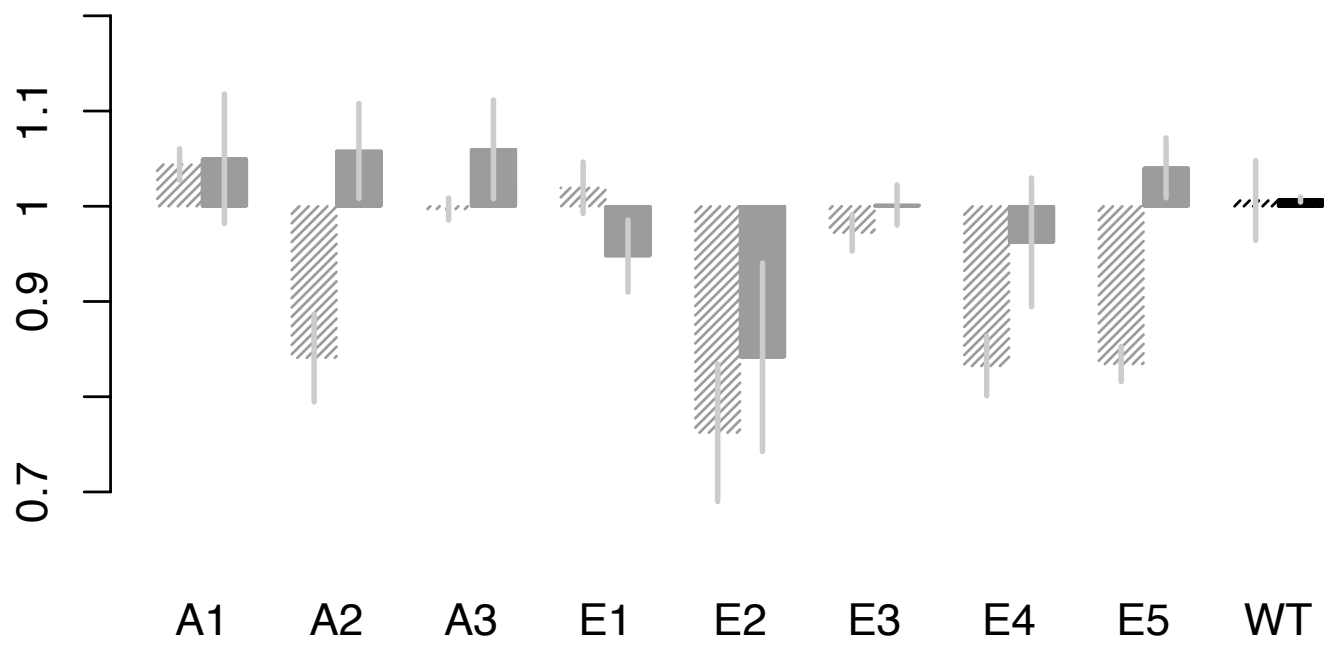

Supplement: Fig. S4 [file rsbl20150361supp4.pdf]
