# Supplementary material for: Plasmid carriage can limit bacteria–phage coevolution
Source: Biol Lett. 2015 Aug;11(8):20150361. doi: 10.1098/rsbl.2015.0361 (PMC4571675; doi:10.1098/rsbl.2015.0361)
Supplement: BL_supplimentary material.docx [file rsbl20150361supp5.docx]

**Fig. S1. No effect of mercury on bacterial growth.** Maximum bacterial growth rates measured in ancestral plasmid containing (+) and plasmid free (-) clones in the absence (dark grey) or presence (light grey) of 8µM HgCl_2_. Bars represent means and error bars show ± 1 standard error (n=6). A 2 way ANOVA found no significant effect of either the plasmid or presence of mercury in the environment (p > 0.05).

**Fig. S2. Population dynamics in the replicate lines of the four treatments;** plasmid free (F, without- and FP, with phage) and plasmid containing (P, without- and PP, with phage). Upper plots: plasmid prevalence estimated by PCR targeting the plasmid encoded loci merA and oriV from 20 colonies per population. Lower plots: Bacterial populations are shown as colour blocks, the height shows bacterial density (log10[cfus ml^-1^]) and colours denote the proportion of non mucoid (blue) and mucoid (red) colonies in the population. The green line shows phage density (log10[pfus ml^-1^]).

**Fig. S3. Reduction in bacterial growth in ancestral plasmid-free and plasmid-carrying strains challenged against the ancestral phage.** Bars show means (n = 6) and lines show standard errors. Both strains are initially susceptible to phage infection with plasmid containing strains having a slightly but significantly higher RBG value at the start of the experiment (t_9.17_ = 11.92, p < 0.001)

**Fig. S4. Effects of plasmid carriage on the costs of phage resistance mutations.** Bars show competitive fitness of eight spontaneous resistance mutants (grey bars) which were isolated against the ancestral (A1-3) or 5 different evolved (E1-5) phages as well as the phage-susceptible wild type strain (WT). Competitive assays were conducted against a marked strain isogenic to the plasmid-free ancestor. Fitness was estimated both in the absence (dashed) and presence (filled) of the plasmid with lines showing standard error.
